# Corrigendum

**DOI:** 10.3892/ol.2013.1360

**Published:** 2013-05-22

**Expression and significance of hypoxia-inducible factor-1α and glucose transporter-1 in laryngeal carcinoma**

XIAO-HONG WU, SU-PING CHEN, JIAN-YING MAO, XUE-XIAN JI, HONG-TIAN YAO and SHUI-HONG ZHOU

Oncology Letters 5: 261–266, 2013; DOI: 10.3892/ol.2013.941

After the publication of the article, the authors detected two errors in their manuscript.

On page 261 in the Abstract, ‘GLUT-1A’ should be ‘GLUT-1’. On page 261 in the Abstract, as well as on page 263, ‘phosphatidylinositol 3-kinase (PI3K)’ should be ‘HIF-1α’.

